# The phase transitions of 4-aminopyridine-based indolocarbazoles: twinning, local- and pseudo-symmetry

**DOI:** 10.1107/S2052520618017341

**Published:** 2019-01-24

**Authors:** Thomas Kader, Berthold Stöger, Johannes Fröhlich, Paul Kautny

**Affiliations:** a Institute of Applied Synthetic Chemistry, TU Wien, Getreidemarkt 9, 1060 Vienna, Austria; b X-Ray Centre, TU Wien, Getreidemarkt 9, 1060 Vienna, Austria

**Keywords:** phase transition, polymorphism, polytypism, twinning, order–disorder (OD) theory

## Abstract

The phase transitions and polymorphism of three 4-aminopyridine-based indolocarbazole analogues are analyzed with respect to symmetry relationships and twinning. Seven polymorphs were structurally characterized using single-crystal diffraction. 5NICz (the indolo[3,2,1-*jk*]carbazole derivative with the C atom in the 5-position replaced by N) crystallizes as a *P*2_1_/*a* high-temperature (270 K) polymorph and as a *Pca*2_1_ low-temperature (150 K) polymorph. Even though their space-group symmetry is not related by a group–subgroup relationship, the local symmetries of both belong to the same order–disorder (OD) groupoid family. Both are polytypes of a maximum degree of order and are twinned by point operations of the other polytype. 2NICz (C atom in the 2-position replaced by N) likewise crystallizes in a high-temperature (*Pcca*, 280 K) polymorph and a low-temperature (*P*2_1_/*c*, 150 K) polymorph. Here, the space-group symmetries are related by a group–subgroup relationship. The low-temperature phase is twinned by the point operations lost on cooling. The crystal structure of bulk 2,5NICz (N-substitution at the 2- and 5-positions) was unrelated to 2NICz and 5NICz and no phase transition was observed. Isolated single crystals of a different polymorph of 2,5NICz, isotypic with 2NICz, were isolated. However, the analogous phase transition in this case takes place at distinctly higher temperatures (> 300 K).

## Abstract 2

The phase transition behaviour and twinning of 4-aminopyridine-based indolocarbazoles are analyzed using the order–disorder theory and group–subgroup relationships.

## 1. Introduction   

Symmetry relationships are crucial in understanding and describing phase transitions (Müller, 2013 ▸). In most cases of displacive phase transition (Tolédanoc *et al.*, 2006 ▸), the symmetry of a high-temperature (HT) phase is a strict super group of the symmetry of the low-temperature (LT) phase (disregarding minor variations of cell parameters). Nevertheless, exceptions exist. For example, numerous incommensurate phases feature a lock-in phase transition to a periodic (and therefore higher-symmetry) LT structure on cooling (Cummins, 1990 ▸). In such a case, both phases are derived from a higher-symmetry prototype structure, which may exist at high temperatures or may be purely hypothetical.

Reconstructive phase transitions are generally not restricted by group–subgroup relationships because, as the name implies, a significant rearrangement of atoms or molecules takes place. There are intermediate cases of symmetry transformations, where modules (layers, rods) are preserved but are arranged differently. In such a case, an interpretation using local symmetry can be insightful.

**Figure 1:**
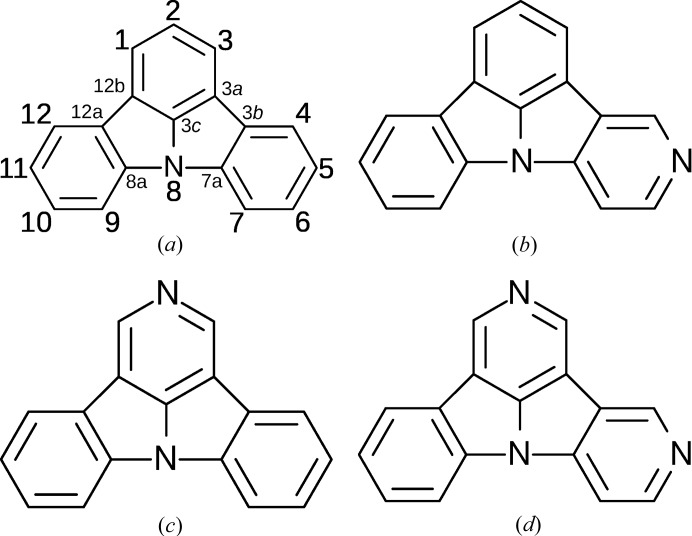
Schematics of (*a*) ICz with IUPAC numbering scheme and (*b*–*d*) the 4-aminopyridine derivatives 5NICz, 2NICz and 2,5NICz.

In this context, we present the structural phase transitions of three 4-aminopyridine derivatives of indolo[3,2,1-*jk*]carbazole (ICz), whereby C atoms *para* to the N atom of ICz are replaced by an N atom. The IUPAC atom numbering-scheme is given in Fig. 1 ▸(*a*). The molecules under investigation, 5NICz, 2NICz and 2,5NICz feature substitution of C atoms by N at the respective positions [Figs. 1 ▸(*b*)–1 ▸(*d*)].

Crystals of 5NICz and 2NICz exist in distinct HT and LT polymorphs, which interconvert below room temperature. 2,5NICz exists in the solid state as three polymorphs. The bulk 2,5NICz-1 crystallizes in a structure unrelated to 5NICz and 2NICz. We could not find any evidence of a phase transition in the solid state for this polymorph. While attempting to obtain improved diffraction data, we found isolated crystals of a different polymorph, which is isostructural to 2NICz. These crystals featured an analogous phase transition (polymorphs designated 2,5NICz-2LT and 2,5NICz-2HT), though with a transition temperature above room temperature.

**Figure 2:**
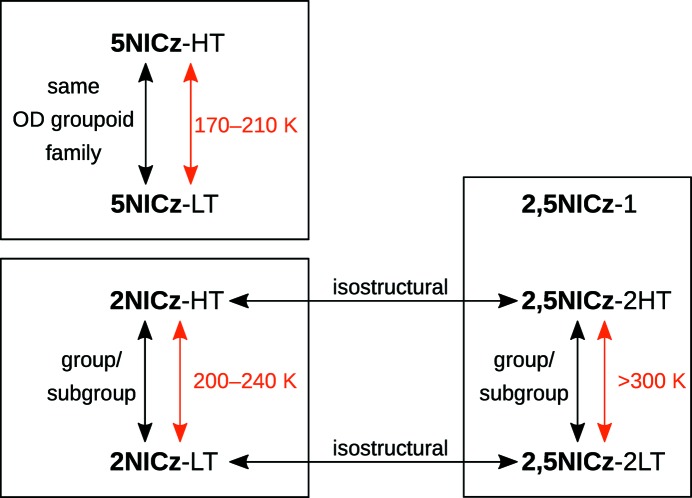
Phase transition temperatures (red arrows) and structural relationships (black arrows) relating the seven polymorphs described in this work. The meaning of ‘same OD groupoid family’ is explained at the end of §3.1.1. Polymorphs not connected by arrows are structurally unrelated.

The observed phase transitions are analyzed with respect to symmetry relationships. Whereas the symmetries of 2NICz-LT and 2NICz-HT polymorphs (and the 2,5NICz-2LT and 2,5NICz-2HT polymorphs) can be described using classical group–subgroup relationships, the local symmetry has to be considered for 5NICz. For this purpose, we use the formalism developed in the framework of order–disorder (OD) theory (Dornberger-Schiff & Grell-Niemann, 1961 ▸; Ferraris *et al.*, 2008 ▸). Despite being of the same name, this theory of polytypism is not related to order–disorder phase transitions. A summary of the phase transitions and the structural relationships between the seven polymorphs is schematized in Fig. 2 ▸.

## 2. Experimental   

### 2.1. Synthesis and crystal growth   

The molecules under investigation were synthesized by ring closure of 9-(2-bromophenyl)-9*H*-carbazole derivatives with the appropriate N-substitution patterns using 5 mol% of an allyl[1,3-bis(2,6-diisopropylphenyl)imidazol-2-ylidene]chloro­palladium(II) catalyst. Reaction optimization studies and full characterizations are given by Kader *et al.* (2019 ▸). Crystals were grown by slow evaporation of acetonitrile solutions.

To prepare 5NICz, a glass vial was charged with 9-(3-bromopyridin-4-yl)-9*H*-carbazole (1 equiv., 324 mg, 1 mmol), K_2_CO_3_ (2 equiv., 276 mg, 2 mmol) and Pd catalyst (0.05 equiv., 29 mg, 0.05 mmol) and flushed with argon. After addition of 10 ml degassed *N*,*N*-dimethylacetamide, the reaction was stirred under argon atmosphere until full conversion. The cooled reaction mixture was poured into water and extracted into CH_2_Cl_2_. The organic phase was dried over Na_2_SO_4_ and concentrated under reduced pressure. The crude product was purified by column chromatography. 2NICz was prepared according to the same procedure starting from 5-(2-bromophenyl)-5*H*-pyrido[4,3-*b*]indole (1 equiv. 322 mg, 1 mmol). Column chromatography afforded fractions of pure 2NICz as well as mixtures of 2NICz and 5NICz. 2,5NICz was prepared according to the same procedure starting from 5-(3-bromopyridin-4-yl)-5*H*-pyrido[4,3-*b*]indole (1 equiv., 324 mg, 1 mmol). 5,11NICz was obtained as secondary product.

### 2.2. Data collection and refinement   

#### 2.2.1. General   

Intensity data were collected in a dry stream of nitrogen on a Bruker Kappa APEX II diffractometer system using graphite-monochromated Mo *K* 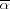 radiation. Data were reduced to intensity values using *SAINT* (Bruker, 2017 ▸). Corrections for absorption and related effects were applied using *SADABS* (Bruker, 2017 ▸). The structures were solved with *SHELXT* (Sheldrick, 2015*a* ▸) and refined with *SHELXL* (Sheldrick, 2015*b* ▸). The atoms were labelled according to IUPAC rules (Fig. 1 ▸ *a*). In the case of two crystallographically different molecules (*Z*′ = 2), prime characters are added for the second molecule. For molecules located on twofold axes, atoms pairs that are equivalent by symmetry are assigned the lower out of the two possible numbers. More data collection and refinement details are summarized in Tables 1 ▸ and 2 ▸, and described in the following sections.

#### 2.2.2. Details for 5NICz   

Crystals of 5NICz were small, yet of reasonable quality according to optical microscopy. Nevertheless, in preliminary scans at the routine temperature of 150 K, all plates featured mediocre reflection quality and diffracted only to small 2θ angles. Such a bad diffraction quality for optically flawless crystals can be a sign of a reconstructive phase transition on cooling. Indeed, crystals cooled to 150 K showed clear signs of fracturing. Two data sets were, therefore, collected with long exposure times, one above the phase transition temperature at 270 K and one after slow cooling to 150 K. To our surprise, even at 270 K the reflection quality was not significantly improved.

For the 150 K data set a reasonable structure solution and refinement, considering the mediocre diffraction quality, was possible in the space group *Pca*2_1_.

The 270 K phase had apparent orthorhombic *C*-centred (*oC*) metrics. But, since a sensible structure solution was not possible in this setting and slight splitting of reflections indicated a lower metric symmetry, the data were reprocessed in the corresponding monoclinic primitive (*mP*) setting. Structure solutions and refinements were performed in the space group 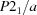 under consideration of twinning by pseudo-merohedry. The non-standard setting of the space group 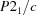 was chosen to ease comparison with the LT polymorph.

#### 2.2.3. Details for 2NICz, 2,5NICz-2LT and 2,5NICz-2HT   

The cell parameters of the LT-polymorph of 2NICz were apparently orthorhombic primitive (*oP*) and, therefore, data were at first processed assuming such a symmetry. A structure solution was successful in the space group *Pccn*. But all attempts at refinements resulted in excessively anisotropic atomic displacement parameters (ADPs) and mediocre residuals. Since, in analogy to 5NICz, reflections at higher diffraction angles were split, an attempt was made in the 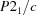 space group under consideration of twinning by pseudo-merohedry. The ADPs as well as the residuals improved significantly (*R*_obs_ > 10% to ∼5.5%). For the HT phase, on the other hand, a refinement using *Pccn* symmetry was successful. In this case, reducing the symmetry to monoclinic did not improve reliability factors.

The cell parameters of 2,5NICz-2 L T suggested a structure isostructural to 2NICz. Refinements were, therefore, performed using starting models derived from the 2NICz model. Even at 300 K, refinements in the LT 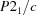 model resulted in significantly improved residuals (*R*_obs_ > 10% to ∼5.3%), even though the metrics are orthorhombic within the estimated standard errors. Only when heating to 380 K was the *Pccn* HT phase clearly observed. In 2,5NICz-2HT, the molecules are located on a twofold axis and the N5 atom is accordingly positionally disordered with the C11 atom in a 1:1 manner. In 2,5NICz-2LT, this position splits in two and both positions were refined as positionally disordered, by constraining the sum of the N-occupancies of both positions to 1. Ultimately, the N-occupancy of one position refined 0.58 (4) (the other accordingly being constrained to 0.42).

#### 2.2.4. Details for 2,5NICz-1   

The structure of 2,5NICz-1 was determined by routine refinement. The 2,5NICz molecule is located on a twofold axis and, therefore, the N5 and C11 atoms are positionally disordered in a 1:1 manner.

### 2.3. X-ray powder diffraction   

Low-temperature X-ray powder diffraction (XRPD) experiments were performed on a Panalytical X’Pert Pro diffractometer equipped with an Oxford Cryosystems PheniX closed cycle cryostat in Bragg–Brentano geometry using Cu *K*α_1,2_ radiation (λ = 1.540598, 1.544426 Å) with an Ni filter and an X’celerator multi-channel detector. The ground bulk sample was placed on an Si single crystal cut along the 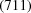 plane. Scans were recorded in vacuum in the 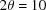
–70° range in 10 K steps from 300 K to 100 K and back to 300 K with heating and cooling rates of 1 K min^−1^ and 5 min isothermals between scans.

## 3. Results and discussion   

### 3.1. The OD polytypism of 5NICz   

#### 3.1.1. Local symmetry   

**Figure 3:**
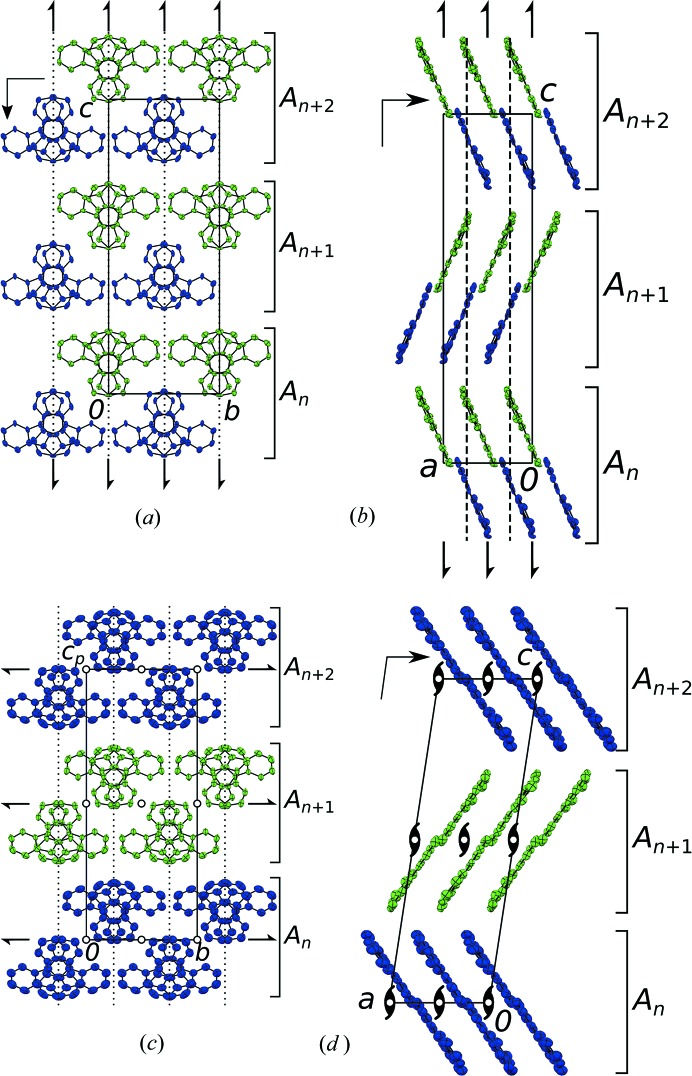
The (*a*,*b*) LT and (*c*,*d*) HT polymorphs of 5NICz viewed down (*a*,*c*) [100] and (*b*,*d*) [010]. Molecules are coloured according to space-group symmetry equivalence. H atoms are omitted for clarity. Layer names are indicated to the right. Crystallographic symmetry elements are indicated by the common graphical symbols (Hahn & Aroyo, 2016 ▸).

**Figure 4:**
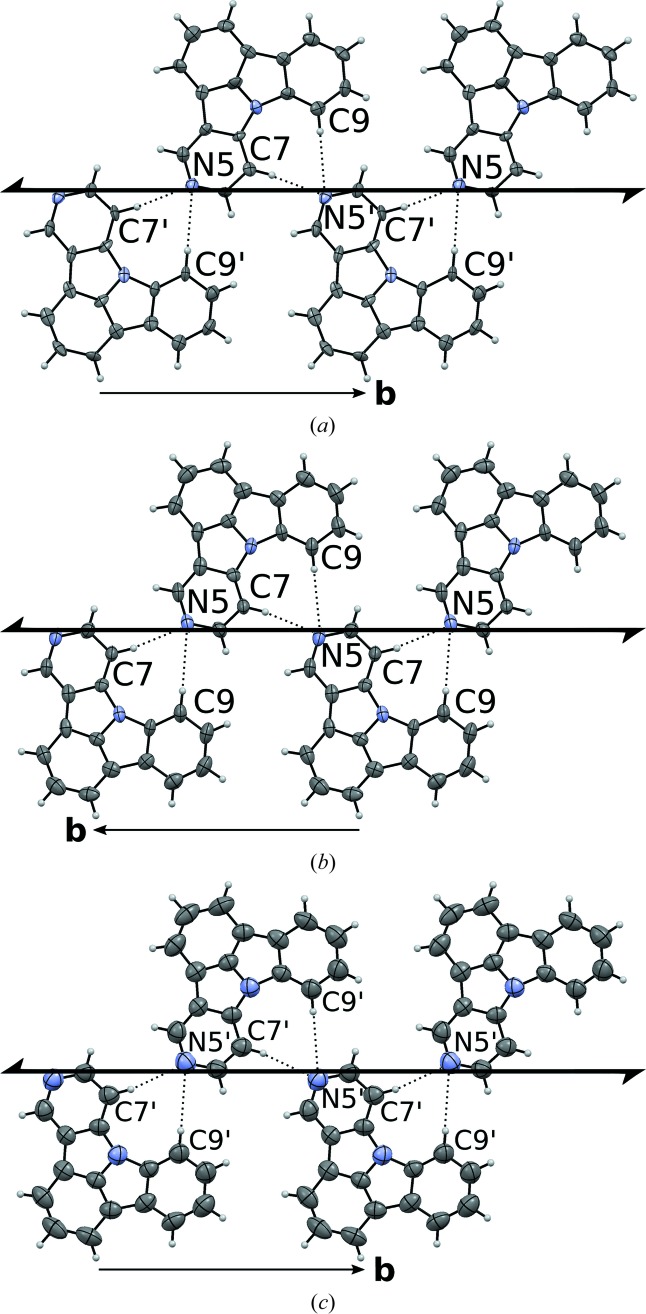
Rods of 5NICz molecules connected by C—H⋯N interactions (dotted lines) extending along [010] in the (*a*) LT and (*b*,*c*) HT polymorphs. Note that in the HT polymorph there are two kinds of rods, whereas in the LT polymorph all rods are related by symmetry. C and N atoms are represented by grey and blue ellipsoids, respectively, drawn at the 50% probability levels, H atoms by white spheres of arbitrary radius. Pseudo (*a*) and crystallographic (*b*,*c*) 2_1_ screw axes are indicated using the usual symbol (Hahn & Aroyo, 2016 ▸).

The HT and LT phases of 5NICz are structurally closely related. They crystallize in the 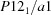 and 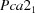 symmetry, respectively and contain *Z*′ = 2 5NICz molecules in the asymmetric unit. The structures can be considered as being composed of *A*_*n*_ layers (*n* is a sequential integer) extending parallel to (001) (Fig. 3 ▸). These layers are made up of rods of molecules which connect via short C—H⋯N contacts (Fig. 4 ▸). Whereas these rods are very similar in both structures (differences will be discussed below), their inclination with respect to the layer plane (001) differs significantly (Fig. 3 ▸). The angles of the least-squares planes of the molecules to the (001) plane are 67.5° and 67.6° versus 55.7° and 56.5° for the LT and HT phases, respectively. Thus, the two kinds of layers can be derived from each other, but they might not be considered as isostructural (Kálmán *et al.*, 1993 ▸) in the strict sense.

Adjacent molecules in the rods described above are related by a 2_1_ operation in the [010] direction. The operation is exact in the HT phase (one crystallographically unique molecule per rod) but only approximate in the LT phase (two molecules per rod). Adjacent rods are, in both polymorphs, related by an *a* operation in the [010] direction. Moreover, they are related by inversions, which is a space group operation in the HT and a local operation in the LT phase. Thus, the layers possess 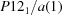 actual (HT) or pseudo (LT) symmetry. Since we will perform an interpretation according to the OD theory, here we use the OD notation of layer symmetry, whereby parentheses indicate the direction lacking translational symmetry (Dornberger-Schiff & Grell-Niemann, 1961 ▸). In the LT polymorph, adjacent *A*_*n*_ layers are related by actual 2_1_ screw rotations in [001] and *c* glide reflections in [100] direction, whereas in the HT polymorph, these are only a pseudo-symmetry operations. In total, both polymorphs are made up of *Z*′ = 2 crystallographically different molecules.

Recognizing the pseudo-symmetry of layers is the key to an OD interpretation. By assuming the pseudo-symmetry to be exact, both polymorphs can be described as members of OD families. The symmetries of OD families are classified into OD groupoid families, which correspond to space group types in classical crystallography (Fichtner, 1979*a* ▸). The symmetries of both polymorphs belong to the same OD groupoid family, which is described by
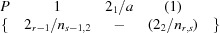
according to the notation of Dornberger-Schiff & Grell-Niemann (1961 ▸). The metric parameter *s* adopts the value *s* = 1 in both cases, which can be expressed by
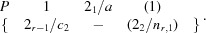
OD groupoids are made up of partial operations (POs), which relate layers but need not apply to the whole stacking sequence. The first line in these symbols gives the symmetry group of the layers [the λ-POs, here 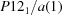]. The second line lists one possible set of operations relating adjacent layers (σ-POs). Since the relative intrinsic translations of the σ-POs are not restricted to those found in space groups, generalized Hermann–Mauguin symbols are used. For example, the 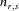 glide reflection in the symbol above has the glide vector 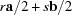. As can be seen in Fig. 3 ▸, the *x*-component of the glide vector is approximately 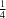 and thus 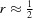 (§3.1.5).

Intrinsic translation components in the stacking direction 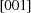 are given with respect to the vector 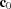, which is perpendicular to the layers and of the length of one layer width. Thus, the 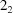 operation in [001] direction has the screw vector 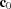 (Fichtner, 1979*b* ▸) since 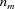 stands for an *n*-fold screw rotation with intrinsic translation of 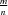 parts of the shortest lattice vector in the translation direction.

#### 3.1.2. Stacking possibilities   

The crucial aspect of OD structures is their ability of crystallizing in different polytypes, which are all locally equivalent (more precisely: pairs of adjacent layers are equivalent). If interactions beyond one layer width and deviations from the prototype layers are neglected, all polytypes can therefore be considered as energetically equivalent. The *NFZ* relationship (Ďurovič, 1997 ▸) is used to derive these stacking possibilities. For 5NICz, there are σ-POs that invert the orientation with respect to the stacking direction (σ-ρ-POs). But owing to 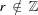 none of these is a reverse continuation, which would mean that it maps 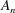 on 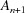 *and* vice versa. In such a case, the NFZ relationship reads as 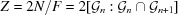, where *Z* is the number of positions 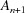 can adopt given 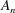 and 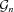 is the group of those 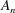 operations that do not invert the orientation with respect to the stacking direction (λ-τ-POs).

Since *s* = 1, the *a* glide planes of all 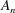 overlap and 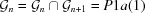. Accordingly, there are 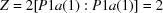 ways of placing 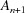 given 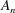. These two possibilities are obtained by applying a 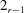 or a 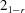 σ-PO on 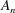, respectively.

#### 3.1.3. Maximum degree of order (MDO) polytypes   

**Figure 5:**
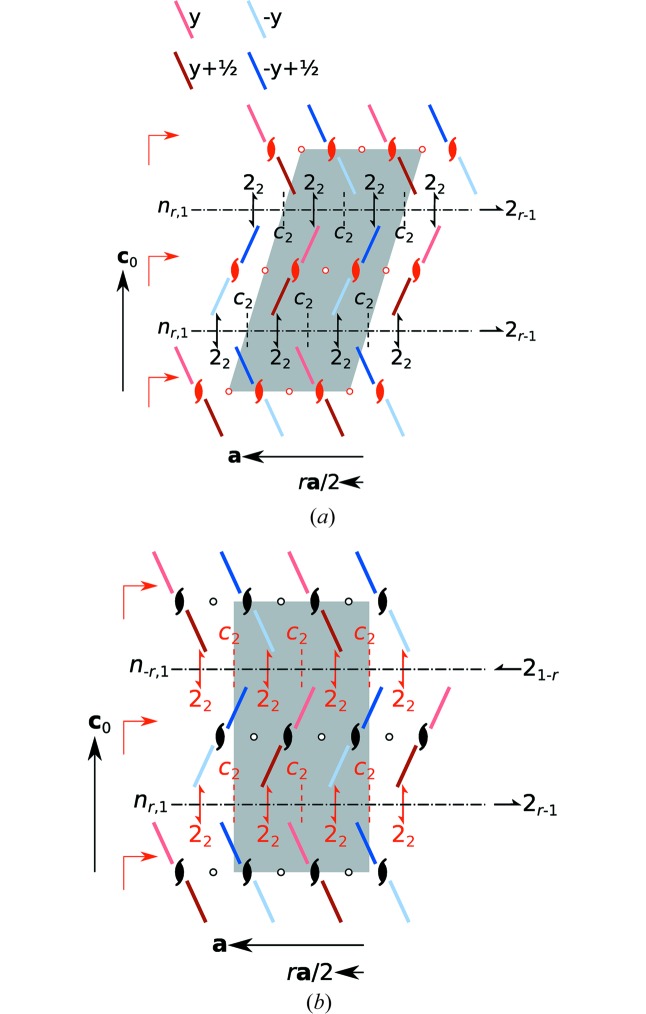
Symmetry of the MDO_1_ and MDO_2_ polytypes of 5NICz viewed along **b**. Molecules are represented by bars which are red on one side and blue on the other. Darker colours indicate translation by **b**/2. λ-POs of the layers and σ-POs relating adjacent layers are indicated by the common graphical symbols (Hahn & Aroyo, 2016 ▸) and, in the case of unusual intrinsic translations, by their printed symbols. Symbols of POs that are valid for the whole polytype are red. The unit cell of the polytypes is marked by a grey backdrop.

Out of the infinity of stacking arrangements, two are of a MDO, which means that they cannot be decomposed into fragments of simpler polytypes (Dornberger-Schiff, 1982 ▸). MDO_1_ [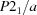, 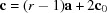] is generated by repeated application of 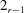 σ-POs; MDO_2_ [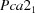, 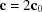] by alternating application of 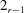 and 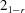 σ-POs. The local symmetry of both polytypes is schematized in Fig. 5 ▸.

In our experience, the overwhelming number of polytypes characterized by single-crystal diffraction is of the MDO kind. Other stacking arrangements may exist at domain interfaces. Indeed, the HT and LT polymorphs of 5NICz are precisely of the MDO_1_ and MDO_2_ type, respectively. Thus, even though the space groups of the two phases are not related by a group–subgroup relationship, their groupoids belong to the same groupoid family with the same restrictions on the metric parameters, *viz.* *s* = 1. Their *local* symmetries are therefore, in a sense, isomorphic, which demonstrates the usefulness of such a symmetry description.

#### 3.1.4. Twinning   

Crystals of OD polytypes are often twinned owing to stacking faults. The possible orientation states of the polytype are derived by coset decomposition of the point group of the polytype in the point group of the OD groupoid family, that is the point group generated by the linear parts of all POs of a polytype. This group is *mmm* for the OD groupoid family of the 5NICz polymorphs.

Thus, MDO_1_ (HT) can appear in [*mmm*:12/*m*1] = 2 orientations, which are related by the operations of the twin law 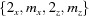. This corresponds precisely to the observed twinning. MDO_2_ (LT) can appear likewise in [*mmm*:*mm*2] = 2 orientations. In this case, the twin law is 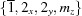. Since the 5NICz molecules possess no significant resonant scatterers under Mo *K*α radiation, this twinning by inversion could not be seen from the diffraction data. Its existence is nevertheless nearly certain. Besides being predicted by OD theory, it is also expected owing to the phase transition from the centro-symmetric MDO_1_ (HT) phase. Point operations lost on phase transformation are typically retained as twin operations.

#### 3.1.5. Desymmetrization and metric parameters   

An important step in assessing an OD model is the quantification of the desymmetrization (Ďurovič, 1979 ▸) compared to the ideal model. Such a desymmetrization is expected (these geometrical differences may stabilize the individual polytypes) but should not be unreasonably large.

In the MDO_1_ (HT) polytype, the symmetry of the actual 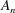 layers is identical to those of the idealized description [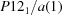]. According to the 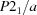 symmetry of the polytypes, the layers are partitioned into two equivalence classes, *viz.* the 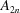 and the 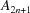 layers. To evaluate the desymmetrization, the 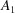 layer was mapped onto the 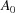 layer by translation of 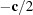 and reflection at the **r**·**a** = 0 plane. The discrepancies between both layers are minute (max: C2/C2′, 0.157 Å), proving the validity of the pseudo-symmetry analysis.

In the MDO_2_ (LT) polytype all layers are related by the *Pca*2_1_ space group symmetry, but the symmetry of the layers is reduced by an index of 2 to 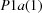. To assess the degree of desymmetrization, the location of the pseudo-2_1_ screw axis was determined by averaging the *x*- and *z*-coordinates of the non-H atoms of the two crystallographically independent molecules. The screw rotation was then applied to a layer. Here, the desymmetrization is even less pronounced than in the HT phase (max: C11/C11′, 0.086 Å).

The metric parameter *r* of the OD groupoids can be derived in the case of MDO_1_ (HT) directly from the cell parameters as 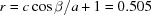. Owing to *r* ≈ 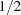, the lattice symmetry of MDO_1_ is pseudo-*oC* and the twinning is by pseudo-merohedry (the reflections of both domains are nearly coincident). More precisely, the twin obliquity calculates from the cell parameters as 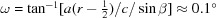. It has to be noted though that the derivation of the cell parameters from single-crystal data is inexact in such a case because overlapping reflections are treated as single reflections during integration. The deviation from *r* = 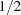 might, therefore, be larger than estimated here.

For MDO_2_ (LT), *r* is derived from the *x*-coordinate of the pseudo-2_1_ operation (see above) as *r* = 4*x* = 0.449. Thus, in both cases, despite the distinctly different orientation of the molecules, the parameter *r* is approximately 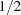.

#### 3.1.6. Structural changes on phase transition   

Even though symmetry considerations are the main focus of this work, changes at the crystallo-chemical level must not be neglected. As has been noted above, the structures of both 5NICz polymorphs are controlled by non-classical C—H⋯N hydrogen interactions, forming chains extending in the [010] direction (Fig. 4 ▸). Each molecule forms a pocket delimited by N8 and the H7 and H9 are in *meta* position to N8. These two H atoms are expected to be the most ‘acidic’ and indeed interact with the N5 lone pair of the adjacent molecule. The hydrogen bonding is distinctly asymmetric with one short (C7⋯N5) and one long (C9⋯N5) interaction (Table 2 ▸).

The C⋯N distances are slightly longer in the HT phase. In return, the C—H⋯N angles are closer to linear, owing to near coplanarity of the connected molecules [Fig. 3 ▸(*d*)]. Overall, the hydrogen bonding can be considered as close to equivalent in both polymorphs.

**Figure 6:**
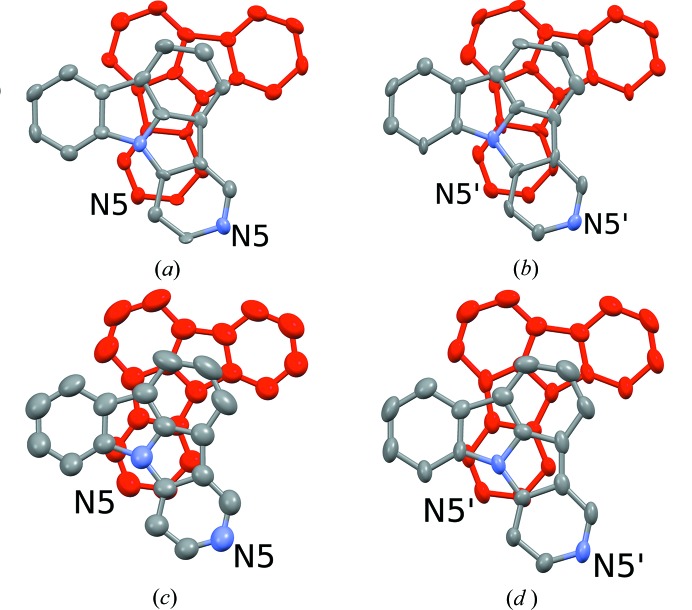
Pairs of 5NICz molecules connected by π–π interactions in the (*a*,*b*) LT and (*c*,*d*) HT polymorphs, projected on the molecular plane. Atom colours of the top molecules as in Fig. 4 ▸; bottom molecules in red for clarity. Ellipsoids are drawn at the 50% probability levels.

Adjacent rods are connected by π–π interactions to layers. Here, the structural changes on phase transition are significant owing to a change in molecule inclination with respect to the layer plane (Fig. 6 ▸). The C—H⋯π contacts relating adjacent layers are, like the hydrogen bonding, very similar in both polymorphs. In summary, the dominant factor in the phase transition seems to be the π–π stacking.

### 3.2. Phase transitions of 2NICz, and 2,5NICz-2LT and 2,5NICz-2HT polymorphs   

#### 3.2.1. Symmetry relationships   

The 2LT and 2HT polymorphs of 2,5NICz are isostructural to the corresponding LT and HT 2NICz polymorphs, whereby the N5 and C11 atoms are positionally disordered. In contrast to 5NICz, the symmetries of the respective HT and LT polymorphs are related by a group–subgroup relationship. As is often observed in such a case, the symmetry of the HT phase (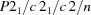, *Z* = 4) is a strict super group (here minimal) of the symmetry of the LT phase (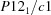, *Z* = 4).

**Figure 7:**
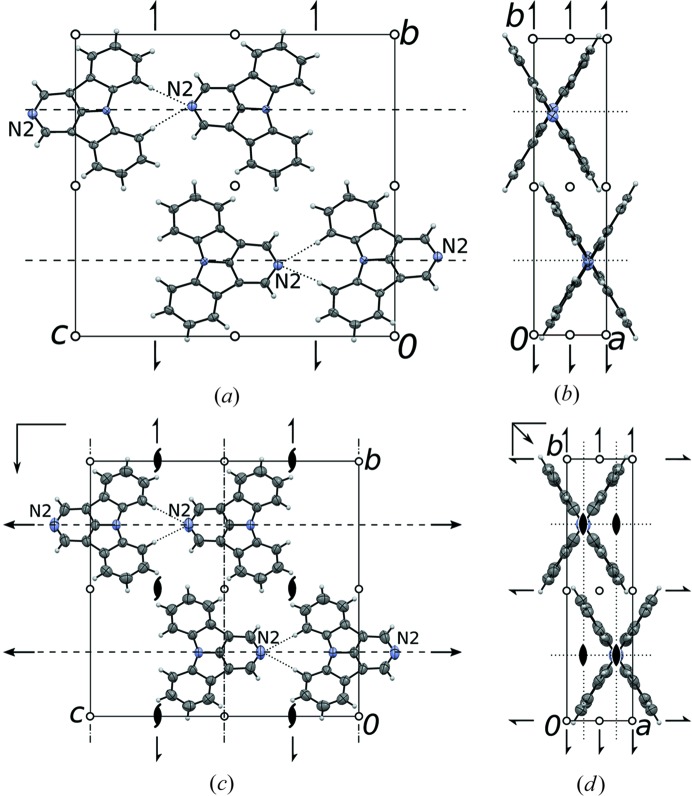
Crystal structures of the (*a*,*b*) LT and (*c*,*d*) HT polymorphs of 2NICz viewed down (*a*,*c*) [100] and (*b*,*d*) [001]. Atom colours as in Fig. 4 ▸. The 2LT and 2HT polymorphs of 2,5NICz are isostructural and not shown. Ellipsoids are drawn at the 50% probability levels. Crystallographic symmetry elements are indicated by the common graphical symbols (Hahn & Aroyo, 2016 ▸).

The structures are again built up of rods of 2NICz (2,5NICz) molecules connected by short C—H⋯N interactions extending along [001] (Fig. 7 ▸). In the HT phase, the molecules are located on a twofold rotation axis and adjacent molecules are related by *c*_[100]_ and *c*_[010]_ glide reflections. The rods, therefore, possess 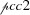 symmetry (Kopsky & Litvin, 2006 ▸). In the [100] direction, adjacent rods are generated by lattice translations. From a thus constructed layer, the final structure with *Pccn* symmetry is generated by 2_1_ screw rotations in the [010] direction.

In the LT phase, the twofold rotation symmetry of the rods is lost. Of the two *c*-glide reflections, only the operation with plane parallel to (010) is retained. Thus, the symmetry of the rods is reduced by an index of 2 from 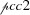 to 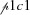. The rods are again related by translations forming layers parallel to (010) and the whole structure then generated by 2_1_ screw rotations in [010] direction, resulting in an overall 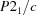 symmetry.

#### 3.2.2. Twinning   

Whereas the HT polymorphs are not twinned, on cooling below the phase transition temperature, the lost point operations are retained as twin operations. The twin law is obtained as a coset of the coset decomposition of the LT in the HT point group. Thus, the LT twin consists of [*mmm*:2/*m*] = 2 domains, whose orientations are related by the operations 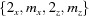. The twinning is by pseudo-merohedry, since the orthorhombic metrics of the lattice are approximately retained. The twin obliquity is derived from the cell parameters as 1.5° (2NICz-LT) and 0.0° (2,5NICz-2LT). Indeed, for 2,5NICz-2LT no splitting of reflections was observed in single-crystal experiments, whereas for 2NICz-LT the twin obliquity is reflected in rows of diverging reflections.

#### 3.2.3. Desymmetrization   

**Figure 8:**
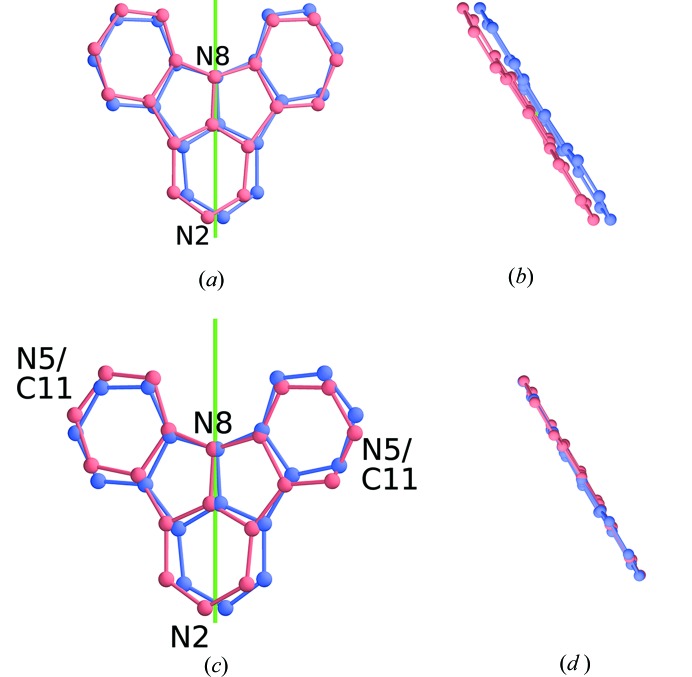
Overlay of molecules and their images by twofold rotation about the (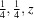) axis in the (*a*,*b*) 2NICz-LT and (*c*,*d*) 2,5NICz-2LT polymorphs viewed down (*a*,*c*) [100] and (*b*,*d*) [001]. The rotation axis is shown in green.

The deviation of β from ideal orthorhombic metrics is a measure of desymmetrization. For a finer evaluation of the desymmetrization, the atomic coordinates were transformed in an orthonormal coordinate system and the pseudo-rotation axis located at 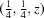 was applied to a molecule. The atoms in the original and the transformed molecule are separated by 0.52–0.68 Å (2NICz-LT) and 0.08–0.53 Å (2,5NICz-2LT). Whereas in 2NICz-LT the deviation is mostly due to a translation away from the rotation axis, in 2,5NICz-2LT the molecules are tilted with respect to the rotation axis of the HT phase (Fig. 8 ▸).

#### 3.2.4. Crystal chemistry   

As in the case of 5NICz, the central crystallo-chemical feature are rods connected by non-classical C—H⋯N hydrogen bonding involving the two H7 and H9 positions. Here, the bonding is more symmetrical, with two equivalent (HT) or only slightly different (by *ca* 0.05 Å; LT) bonds (Table 3 ▸). Enlarged ADPs of the N2 atom (Fig. 7 ▸ *c*) indicate that the desymmetrization is dynamic, *i.e.* the orientations of the molecules oscillate between the two possible asymmetric states. Since the remaining structural changes are likewise minute, one can assume that the desymmetrization of the hydrogen-bonding is the decisive factor in the phase transition. Numerous reported solid–solid phase transitions are due to such a dynamic desymmetrization, a classical example being the KH_2_PO_4_ (KDP) family of ferroelectrics (Peercy, 1975 ▸).

### 3.3. 2,5NICz-1   

**Figure 9:**
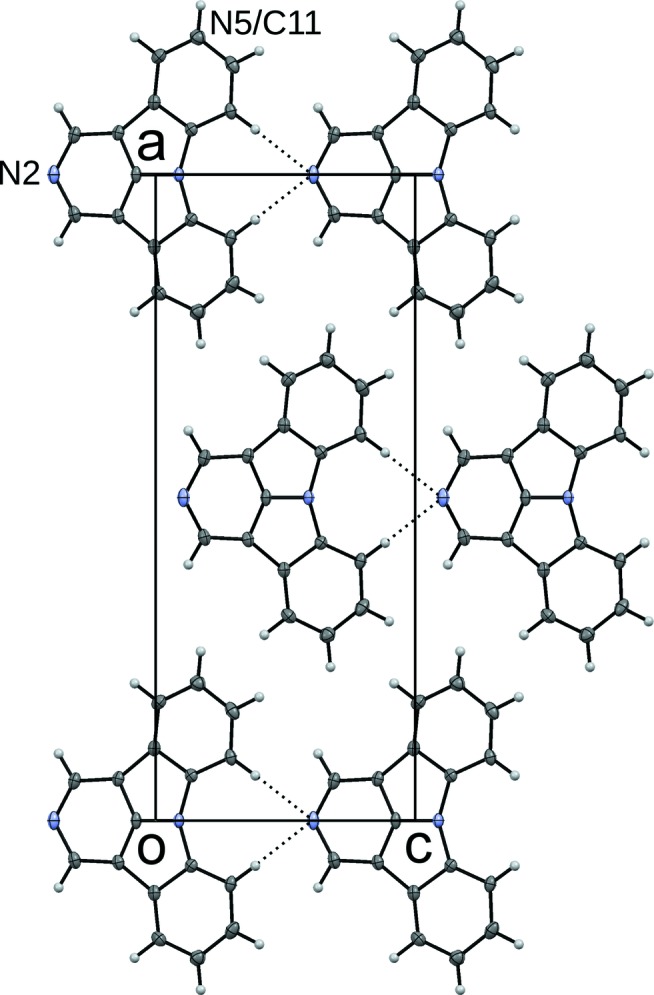
The crystal structure of 2,5NICz-1 viewed down [010]. Atom colours as in Fig. 4.

The bulk polymorph 2,5NICz-1 features a crystallographically non-challenging structure with *Pmn*2_1_ symmetry. In analogy to the other structures presented here, the 2,5NICz molecules are connected by hydrogen bonds to chains (Fig. 9 ▸). In contrast to the 2,5NICz-2LT and 2,5NICz-2HT polymorphs, the connected molecules are coplanar (related by a **b** + **c** lattice translation), demonstrating that the inclination is determined by packing effects.

### 3.4. Powder diffraction   

**Figure 10:**
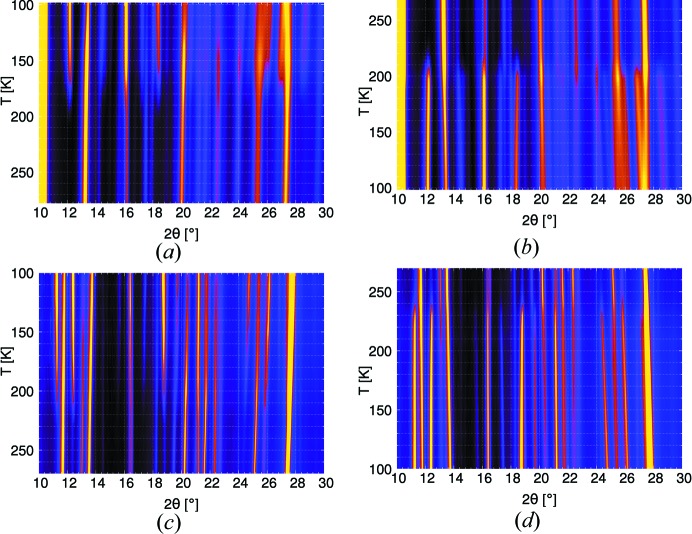
Low-temperature XRPD scans of 5NICz (*a*,*b*) and 2NICz (*c*,*d*) over the 2θ range 10–30° on heating (*a*,*c*) and cooling (*b*,*d*) shown as heat maps. Maximum and minimum intensities are yellow and black, respectively.

To determine the stability ranges of the LT and HT polymorphs and to rule out additional phase transitions, powdered samples of 5NICz and 2NICz were subjected to low-temperature powder diffraction (Fig. 10 ▸). In a bulk sample of 2,5NICz only the orthorhombic polymorph 1 could be seen by X-ray diffraction, which does not possess a phase transition in the solid state. Thus, in this case the exact phase transition temperature could not be determined. In both cases, 5NICz and 2NICz, the HT
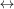
LT transitions are clearly showed by appearance/vanishing of peaks and a distinct hysteresis of ∼20 K is observed [5NICz: transitions at 180–170 K (cooling) versus  200–210 K (heating); 2NICz: 210–200 K (cooling) versus 230–240 K (heating)]. No other phase transitions are apparent. The hysteresis suggests a phase transition of the first order. Even though neither powder diffraction nor DSC data for the 2,5NICz-2 polymorph could be acquired, experiments on the single crystal showed a smooth transition to the orthorhombic phase. This phase transition might be, therefore, of the second order.

## 4. Conclusion   

From a crystallo-chemical point of view, the polymorphs of 5NICz, 2NICz and 2,5NICz are all closely related. Their structures are determined by non-classical C—H⋯N hydrogen bonding. The molecular orientations in the thus-formed rods differ owing to either N-substitution at different rings or with respect to the rotation of adjacent molecules.

Nevertheless, they are fundamentally different from a crystallographical point of view. The transitions between 2NICz-LT and 2NICz-HT, and the isostructural 2,5NICz-2LT and 2,5NICz-2HT are clearly displacive and, as expected in such a case, the symmetries of the polymorphs are related by a group–subgroup relationship. The transition of 5NICz, on the other hand, is a borderline case between displacive and reconstructive, with layers that are in principle similar but feature distinctly changed inclination of the molecules. More interestingly, the symmetry relationship between both polymorphs can only be understood by analysis of their space groupoids in the sense of OD theory. Thus, it is shown that a unified theory of local symmetry is needed.

## Supplementary Material

Crystal structure: contains datablock(s) global, 2NICz-LT, 2NICz-HT, 5NICz-LT, 5NICz-HT, 25NICz-1, 25NICz-2LT, 2NICz-2HT. DOI: 10.1107/S2052520618017341/wf5145sup1.cif

Structure factors: contains datablock(s) 2NICz-LT. DOI: 10.1107/S2052520618017341/wf51452NICz-LTsup2.hkl

Structure factors: contains datablock(s) 2NICz-HT. DOI: 10.1107/S2052520618017341/wf51452NICz-HTsup3.hkl

Structure factors: contains datablock(s) 5NICz-LT. DOI: 10.1107/S2052520618017341/wf51455NICz-LTsup4.hkl

Structure factors: contains datablock(s) 5NICz-HT. DOI: 10.1107/S2052520618017341/wf51455NICz-HTsup5.hkl

Structure factors: contains datablock(s) 25NICz-1. DOI: 10.1107/S2052520618017341/wf514525NICz-1sup6.hkl

Structure factors: contains datablock(s) 25NICz-2LT. DOI: 10.1107/S2052520618017341/wf514525NICz-2LTsup7.hkl

Structure factors: contains datablock(s) 2NICz-2HT. DOI: 10.1107/S2052520618017341/wf51452NICz-2HTsup8.hkl

CCDC references: 1883688, 1883689, 1883690, 1883691, 1883692, 1883693, 1883694

## Figures and Tables

**Table:** For all structures: colourless crystals, Mo *K*α radiation, Bruker Kappa APEX II CCD diffractometer, multi-scan absorption correction, H-atom parameters constrained.

	5NICz-LT	5NICz-HT	2NICz-LT	2NICz-HT
Crystal data				
Chemical formula	C_17_H_10_N_2_	C_17_H_10_N_2_	C_17_H_10_N_2_	C_17_H_10_N_2_
*M*_r_	242.27	242.27	242.27	242.27
Crystal system, space group	Orthorhombic, *P* *c* *a*2_1_	Monoclinic, *P*2_1_/*a*	Monoclinic, *P*2_1_/*c*	Orthorhombic, *P* *c* *c* *n*
Temperature (K)	150	270	150	280
*a*, *b*, *c* (Å)	7.394 (9), 10.953 (13), 29.06 (3)	8.2204 (15), 10.898 (2), 26.905 (5)	4.064 (8), 16.58 (3), 17.57 (3)	4.1239 (8), 16.404 (3), 17.252 (3)
α, β, γ (°)	90, 90, 90	90, 98.691 (4), 90	90, 91.53 (5), 90	90, 90, 90
*V* (Å^3^)	2353 (5)	2382.6 (8)	1184 (4)	1167.1 (4)
*Z*	8	8	4	4
μ (mm^−1^)	0.08	0.08	0.08	0.08
Crystal shape	Rod	Rod	Rod	Rod
Crystal size (mm)	0.55 × 0.16 × 0.10	0.55 × 0.16 × 0.10	0.55 × 0.08 × 0.04	0.30 × 0.10 × 0.06

Data collection				
*T*_min_, *T*_max_	0.552, 0.745	0.600, 0.746	0.549, 0.746	0.569, 0.746
No. of measured, independent and observed [*I* > 2σ(*I*)] reflections	11 809, 3757, 2598	36 052, 5710, 3042	10 904, 2763, 1908	12 863, 1407, 778
*R*_int_	0.075	0.077	0.063	0.061

Refinement				
*R*[*F* ^2^ > 2σ(*F* ^2^)]	0.098	0.091	0.055	0.043
*wR*[*F* ^2^ > 2σ(*F* ^2^)]	0.242	0.223	0.110	0.093
*R*(all)	0.134	0.168	0.098	0.099
*wR*(all)	0.276	0.280	0.128	0.119
*S*	1.03	1.06	1.02	1.00
No. of reflections	3757	5710	2763	1407
No. of parameters	343	344	173	88
No. of restraints	1	0	0	0
Δρ_max_, Δρ_min_ (e Å^−3^)	0.65, −0.33	0.50, −0.30	0.20, −0.27	0.17, −0.18
Absolute structure	?†	–	–	–
Absolute structure parameter	?†	–	–	–
Twin operation	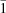	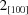	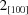	–
Volume fraction (%)	?†	53.9: 46.1 (3)	53.3: 46.7 (3)	–

**Table:** 

	2,5NICz-1	2,5NICz-2LT	2NICz-2HT
Crystal data			
Chemical formula	C_16_H_9_N_3_	C_16_H_9_N_3_	C_16_H_9_N_3_
*M*_r_	243.26	243.26	243.26
Crystal system, space group	Orthorhombic, *P* *m* *n*2_1_	Monoclinic, *P*2_1_/*c*	Orthorhombic, *P* *c* *c* *n*
Temperature (K)	100	300	380
*a*, *b*, *c* (Å)	19.316 (4), 3.7013 (8), 7.7676 (18)	4.0049 (13), 16.518 (5), 17.179 (5)	4.0741 (8), 16.416 (3), 17.230 (3)
α, β, γ (°)	90, 90, 90	90, 90.007 (10), 90	90, 90, 90
*V* (Å^3^)	555.3 (2)	1136.5 (6)	1152.3 (4)
*Z*	2	4	4
μ (mm^−1^)	0.09	0.09	0.09
Crystal shape	Plate	Plate	Plate
Crystal size (mm)	0.45 × 0.23 × 0.03	0.32 × 0.10 × 0.02	0.32 × 0.10 × 0.02

Data collection			
*T*_min_, *T*_max_	0.424, 0.493	0.513, 0.745	0.609, 0.745
No. of measured, independent and observed [*I* > 2σ(*I*)] reflections	5744, 1608, 1458	8439, 2027, 997	2369, 971, 393
*R*_int_	0.034	0.088	0.056

Refinement			
*R*[*F* ^2^ > 2σ(*F* ^2^)]	0.041	0.053	0.050
*wR*[*F* ^2^ > 2σ(*F* ^2^)]	0.100	0.106	0.108
*R*(all)	0.045	0.150	0.158
*wR*(all)	0.104	0.147	0.152
*S*	1.07	0.96	0.93
No. of reflections	1608	2027	971
No. of parameters	91	174	88
No. of restraints	1	0	0
Δρ_max_, Δρ_min_ (e Å^−3^)	0.33, −0.27	0.25, −0.23	0.13, −0.18
Absolute structure	Flack *x* determined using 561 quotients [(I+)−(I−)]/[(I+)+(I−)] (Parsons *et al.*, 2013 ▸)	–	–
Absolute structure parameter	−0.3 (10)	–	–
Twin operation	?†	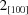	–
Volume fraction (%)	?†	54.7:45.3 (4)	–

Computer programs: *SAINT*, *APEX*II, *SADABS* (Bruker, 2017 ▸), *SHELXL*2014/7 (Sheldrick, 2015*b* ▸).

† Not determined owing to a lack of significant resonant scatterers.

**Table 2:** Non-classical C—H⋯N hydrogen bonding in both polymorphs of 5NICz

*D*—H⋯*A*	*D*—H (Å)	H⋯*A* (Å)	*D*⋯*A* (Å)	*D*—H⋯*A* (°)
150 K				
C7—H⋯N5′	0.95	2.47	3.389 (14)	162.7
C9—H⋯N5′	0.95	2.80	3.722 (14)	163.2
C7′—H⋯N5	0.95	2.51	3.426 (14)	160.7
C9′—H⋯N5	0.95	2.78	3.706 (15)	163.8
270 K				
C7—H⋯N5	0.93	2.53	3.446 (9)	167.0
C9—H⋯N5	0.93	2.86	3.784 (9)	173.8
C7′—H⋯N5′	0.93	2.50	3.404 (5)	165.0
C9′—H⋯N5′	0.93	2.87	3.789 (7)	172.1

**Table 3:** Non-classical C—H⋯N hydrogen bonding in the HT and LT polymorphs of 2NICz and the 2HT and 2LT polymorphs of 2,5NICz

*D*—H⋯*A*	*D*—H (Å)	H⋯*A* (Å)	*D*⋯*A* (Å)	*D*—H⋯*A* (°)
2NICz-LT				
C7—H7⋯N2	0.95	2.59	3.540 (6)	174.5
C9—H9⋯N2	0.95	2.65	3.594 (6)	174.7
2NICz-HT				
C7—H7⋯N2 (2×)	0.93	2.60	3.532 (2)	175.6
2,5NICz-2LT				
C7—H7⋯N2	0.93	2.58	3.505 (6)	177.7
C9—H9⋯N2	0.93	2.60	3.529 (6)	175.5
2,5NICz-2HT				
C7—H7⋯N2 (2×)	0.93	2.61	3.538 (5)	175.7
